# RETRACTION: Comparative Analysis of Wound Healing Techniques in Postoperative Bladder Cancer Patients

**DOI:** 10.1111/iwj.70541

**Published:** 2025-04-18

## Abstract

**RETRACTION:** ZhongL. and WangF. , “Comparative Analysis of Wound Healing Techniques in Postoperative Bladder Cancer Patients,” International Wound Journal 21, no. 3 (2024): e14820, 10.1111/iwj.14820.38425151 PMC10904970

The above article, published online on 29 February 2024, in Wiley Online Library (http://onlinelibrary.wiley.com/), has been retracted by agreement between the journal Editor in Chief, Professor Keith Harding; and John Wiley & Sons Ltd. Following an investigation by the publisher, all parties have concluded that this article was accepted solely on the basis of a compromised peer review process. The editors have therefore decided to retract the article. The authors did not respond to our notice regarding the retraction.

**RETRACTION:** L. Zhong and F. Wang , “Comparative Analysis of Wound Healing Techniques in Postoperative Bladder Cancer Patients,” International Wound Journal 21, no. 3 (2024): e14820, 10.1111/iwj.14820.38425151 PMC10904970

The above article, published online on 29 February 2024, in Wiley Online Library (http://onlinelibrary.wiley.com/), has been retracted by agreement between the journal Editor in Chief, Professor Keith Harding; and John Wiley & Sons Ltd. Following an investigation by the publisher, all parties have concluded that this article was accepted solely on the basis of a compromised peer review process. The editors have therefore decided to retract the article. The authors did not respond to our notice regarding the retraction.

